# Precision Assessment of COVID-19 Phenotypes Using Large-Scale Clinic Visit Audio Recordings: Harnessing the Power of Patient Voice

**DOI:** 10.2196/20545

**Published:** 2021-02-19

**Authors:** Paul J Barr, James Ryan, Nicholas C Jacobson

**Affiliations:** 1 The Dartmouth Institute for Health Policy & Clinical Practice Geisel School of Medicine at Dartmouth College Lebanon, NH United States; 2 The Center for Technology and Behavioral Health Geisel School of Medicine at Dartmouth College Lebanon, NH United States; 3 Ryan Family Practice Ludington, MI United States; 4 Biomedical Data Science Geisel School of Medicine at Dartmouth College Lebanon, NH United States; 5 Department of Psychiatry Geisel School of Medicine at Dartmouth and Dartmouth Hitchcock Health Lebanon, NH United States

**Keywords:** communication, coronavirus, COVID-19, Machine Learning, natural language processing, patient-physician communication, patient records, recording

## Abstract

COVID-19 cases are exponentially increasing worldwide; however, its clinical phenotype remains unclear. Natural language processing (NLP) and machine learning approaches may yield key methods to rapidly identify individuals at a high risk of COVID-19 and to understand key symptoms upon clinical manifestation and presentation. Data on such symptoms may not be accurately synthesized into patient records owing to the pressing need to treat patients in overburdened health care settings. In this scenario, clinicians may focus on documenting widely reported symptoms that indicate a confirmed diagnosis of COVID-19, albeit at the expense of infrequently reported symptoms. While NLP solutions can play a key role in generating clinical phenotypes of COVID-19, they are limited by the resulting limitations in data from electronic health records (EHRs). A comprehensive record of clinic visits is required—audio recordings may be the answer. A recording of clinic visits represents a more comprehensive record of patient-reported symptoms. If done at scale, a combination of data from the EHR and recordings of clinic visits can be used to power NLP and machine learning models, thus rapidly generating a clinical phenotype of COVID-19. We propose the generation of a pipeline extending from audio or video recordings of clinic visits to establish a model that factors in clinical symptoms and predict COVID-19 incidence. With vast amounts of available data, we believe that a prediction model can be rapidly developed to promote the accurate screening of individuals at a high risk of COVID-19 and to identify patient characteristics that predict a greater risk of a more severe infection. If clinical encounters are recorded and our NLP model is adequately refined, benchtop virologic findings would be better informed. While clinic visit recordings are not the panacea for this pandemic, they are a low-cost option with many potential benefits, which have recently begun to be explored.

## Challenges in Identifying COVID-19 Clinical Phenotypes

COVID-19 cases are exponentially increasing worldwide; however, clinical COVID-19 phenotypes remain unclear. A clinical phenotype is an observable characteristic (ie, symptom) of a disease in a particular individual. A meta-analysis of COVID-19–related symptom presentations reported that the most frequent clinical symptoms are fever, cough, fatigue, and dyspnea [1]. However, the meta-analysis reported considerable heterogeneity (*I*^2^=84.9%-96.4%) among studies, potentially suggestive of the extreme heterogeneity among these symptoms at the individual patient level. Other less frequent COVID-19 symptoms include anosmia, dysgeusia, headache, sore throat, rhinorrhea, diarrhea, nausea, and myalgias [2]. However, their clinical implication, prevalence, and importance remain unclear.

A traditional reductionist approach to identifying COVID-19 treatments is not as simple as extrapolating the current knowledge toward our limited SARS-CoV-2 model. Clinical treatments are often based on a set of established biochemical markers, and reports of less frequent symptoms of a disease may reveal a biochemical pathway that can be subjected to pharmacotherapeutic intervention with previously unreported agents. Only laboratory tests can confirm a diagnosis of COVID-19, but such tests are in short supply. This presents an unprecedented need to develop better assessment methods to identify and generate heterogeneity in the clinical profile of COVID-19 and other viral diseases across the entire health care system. The urgency of this need cannot be understated, as it holds a key to understand how to identify and treat COVID-19 more accurately.

## Using “Big Data” to Understand the Clinical Manifestations of COVID-19

Natural language processing (NLP) and machine learning may yield a method to rapidly identify individuals at a high risk for COVID-19 and to understand key symptoms upon clinical manifestation and presentation [3]. The existing applications of NLP and machine learning in medical diagnostics are based on a combination of structured (eg, symptom codes, medications, laboratory findings, etc) and unstructured (eg, visit notes, radiology reports, etc) data recorded by clinicians in patients’ electronic health records (EHRs). Using NLP and machine learning approaches, data on documented signs and symptoms in the EHR are already being used to identify clinical conditions (computational phenotyping) [4]. Such NLP-based efforts are currently being applied to unstructured text data captured in the EHR from telehealth consultations to develop better screening tools for COVID-19 [5]. Ancillary data can improve the accuracy of computational phenotyping, such as information from disease registries. However, the performance of any model is determined by the quality of data used to generate it, and concerns exist about the fullness of data captured in the EHR.

## Limitations of EHR Data

This considerable degree of symptom heterogeneity reported among patients with COVID-19 can deter the accurate documentation of less frequently reported symptoms in the EHR. Documentation inaccuracies in electronic medical records are not a new phenomenon; an analysis of data from 105 clinics indicated that 90% of clinician notes had at least one error, including 636 documentation errors that accounted for 181 charted findings that did not take place and 455 findings that were not charted [6]. Data on such symptoms may not be accurately synthesized into patient records owing to the pressing need to treat patients in overburdened health care settings. In this scenario, clinicians may focus on documenting widely reported symptoms that suggest a diagnosis of COVID-19 albeit at the expense of infrequently reported symptoms because overburdened clinicians are more likely to be affected by cognitive biases such as anchoring and confirmation biases [7]. Additionally, codes of the International Classification of Diseases (10th revision), the mainstay of documentation in electronic medical records, do not adequately capture COVID-19–related symptoms [8]. While NLP solutions can play a key role in generating clinical phenotypes of COVID-19, they are limited by the resulting limitations in EHR data. A comprehensive record of the clinic visits is required—an audio recording may be the solution [9].

## Clinical Phenotypes Based on Audio Recordings of Clinic Visits

A small but growing number of health systems routinely obtain audio recordings, and, in some cases, video recordings of clinic visits [9,10]. For example, human scribes are commonly employed to review recordings of clinic visits and make detailed notes, thus reducing the documentation burden on clinicians and improving the accuracy of data entered in the EHR. A recording of the clinic visit represents a more comprehensive and accurate record of patient-reported symptoms. If performed at scale, a combination of data from the EHR and recordings of clinic visits can be used to power NLP and machine learning models, thus rapidly generating a clinical phenotype of COVID-19 and infections with subsequent SARS-CoV-2 strains. In addition to a more comprehensive record of symptoms discussed, recordings also asynchronously collect additional ancillary information such as the type and frequency of cough, which can help improve the precision of phenotyping.

The generation of NLP and machine learning models requires the transcription of vast quantities of conversations of patients being investigated for COVID-19 upon clinic visits (with subsequent confirmatory laboratory tests for the disease) and the annotation of these transcripts by annotators trained to identify symptom mentions. The performance of automated speech recognition algorithms has significantly improved [10], allowing for the real-time use of audio data rather than transcripts of audio data, which are more time-consuming to obtain. Real-time risk assessment is critical when responding to an infectious disease such as COVID-19, since it allows for individuals to identify their risk level and more rapidly self-isolate, thus reducing the risk of disease transmission. Data annotation to generate models that can accurately identify symptoms is not without its challenges, many of which have been summarized by Quiroz et al [11]. It can be difficult for annotators to identify vaguely indicated symptoms from the unstructured natural language used in clinic visit conversations, with a negative impact on model performance. Rigorous training of annotators can help mitigate this challenge; however, such training and annotation is time-consuming and would require a large team of annotators to rapidly meet the immediate need for such an analysis. In addition, model training requires human input and time. Furthermore, the generation of optimal data would require continuous data refinement, wherein records of suspected cases are replaced by the findings of confirmatory tests so as not to correspond to clinician views or biases.

## Implications of the Adoption of Clinic Visit Recordings in Managing COVID-19

**Figure 1:**
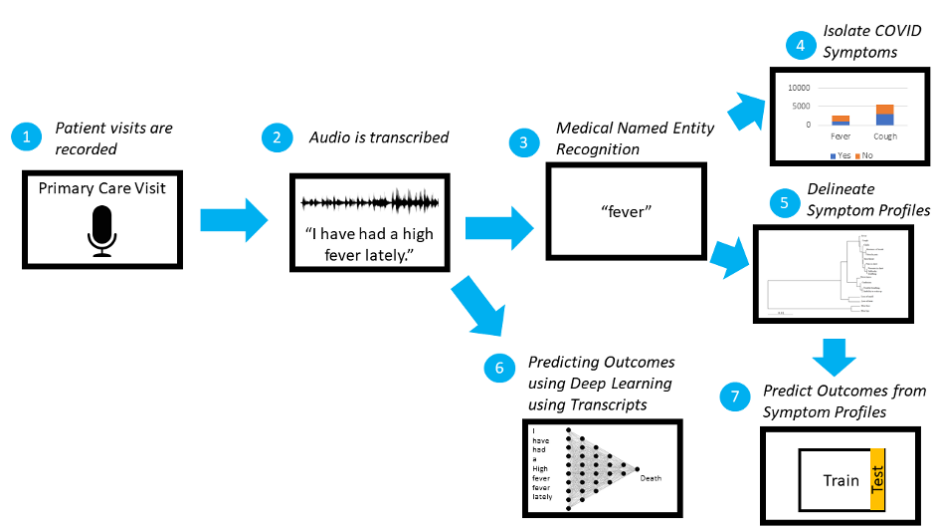
Natural language processing pipeline from audio recordings to the establishment of a clinical phenotype of COVID-19.

We propose the generation of a pipeline from the audio recordings of clinic visits to models based on clinical symptoms and the prediction of COVID-19 incidence (Figure 1). With vast amounts of available data, we believe a prediction model can be rapidly developed to promote accurate screening of individuals at risk of COVID-19. Beyond the challenge of generating a clinical phenotype, an unfiltered account of a patient’s clinical experience of the disease allows us to answer other pressing questions, such as those related to understanding the constellation of patient characteristics that may predict a greater risk of a more severe infection. If clinical consultations are recorded and our NLP model is adequately refined, benchtop virologic findings are better informed. Recordings of clinic visits also provide a historic reference, such that we may be better prepared for subsequent pandemics.

With the mass transition to telehealth consultations and the availability of guidance for conducting remote assessments of COVID-19 via telehealth at primary care centers [12], an opportunity to capture audio recordings of consultations at scale is now available. An accurate model predicting a higher risk of COVID-19 could be applied to telehealth consultations with the added benefit of reducing the exposure risk among clinicians, patients, and the general public. The use of NLP for remote COVID-19 screening is already emerging; for example, audio recordings of cough sounds are being used to identify individuals with COVID-19 [13,14].

## Data From Beyond the Clinic

While recordings of clinic visits are not the panacea for this pandemic, they are a low-cost alternative with many potential benefits that have recently begun to be explored. Beyond audio recordings, video recordings of telehealth consultations can provide additional diagnostic information such as skin appearance [12]. At-home voice-based technologies such as Amazon Alexa, Apple’s Siri, and Google Home can also be used, allowing further information from outside of clinic visits to supplement predictive models [15]. For example, the Mayo Clinic has recently added a skill to Amazon Alexa called “Answers on COVID-19,” which provides resources on COVID-19 and a virtual questionnaire to determine a person's symptoms and whether the person should get tested for COVID-19 [16].

Considering current accelerated efforts to manage COVID-19, care must be taken to rigorously protect sensitive data, with existing challenges in accessing the corpus of patient recordings needed to generate these models [11]. A data collection method should only be used entirely with an opt-in voluntary framework to preserve privacy and confidentiality; however, this method can help obtain data on COVID-19 symptom exacerbation at a scale unattainable with all traditional methods. This, as is often the case, points toward an evolving learning health system capable of managing computable knowledge.

## Abbreviations

EHR: electronic health record
NLP: natural language processing

## References

[1] Yang J, Zheng Y, Gou X, Pu K, Chen Z, Guo Q, Ji R, Wang H, Wang Y, Zhou Y (2020). Prevalence of comorbidities and its effects in patients infected with SARS-CoV-2: a systematic review and meta-analysis. Int J Infect Dis.

[2] McIntosh K, Hirsch M, Bloom A Coronavirus disease 2019 (COVID-19). UpToDate.

[3] Liao KP, Cai T, Savova GK, Murphy SN, Karlson EW, Ananthakrishnan AN, Gainer VS, Shaw SY, Xia Z, Szolovits P, Churchill S, Kohane I (2015). Development of phenotype algorithms using electronic medical records and incorporating natural language processing. BMJ.

[4] Richesson RL, Hammond WE, Nahm M, Wixted D, Simon GE, Robinson JG, Bauck AE, Cifelli D, Smerek MM, Dickerson J, Laws RL, Madigan RA, Rusincovitch SA, Kluchar C, Califf RM (2013). Electronic health records based phenotyping in next-generation clinical trials: a perspective from the NIH Health Care Systems Collaboratory. J Am Med Inform Assoc.

[5] Obeid J, Davis M, Turner M, Meystre S, Heider P, O'Bryan Edward C, Lenert Leslie A (2020). An artificial intelligence approach to COVID-19 infection risk assessment in virtual visits: A case report. J Am Med Inform Assoc.

[6] Weiner S, Wang S, Kelly B, Sharma G, Schwartz A (2020). How accurate is the medical record? A comparison of the physician's note with a concealed audio recording in unannounced standardized patient encounters. J Am Med Inform Assoc.

[7] Yuen T, Derenge D, Kalman N (2018). Cognitive bias: Its influence on clinical diagnosis. J Fam Pract.

[8] Crabb B, Lyons A, Bale M, Martin Valerie, Berger Ben, Mann Sara, West William B, Brown Alyssa, Peacock Jordan B, Leung Daniel T, Shah Rashmee U (2020). Comparison of International Classification of Diseases and Related Health Problems, Tenth Revision Codes With Electronic Medical Records Among Patients With Symptoms of Coronavirus Disease 2019. JAMA Netw Open.

[9] Elwyn G, Barr PJ, Piper S (2018). Digital clinical encounters. BMJ.

[10] Barr PJ, Bonasia K, Verma K, Dannenberg MD, Yi C, Andrews E, Palm M, Cavanaugh KL, Masel M, Durand M (2018). Audio-/Videorecording Clinic Visits for Patient's Personal Use in the United States: Cross-Sectional Survey. J Med Internet Res.

[11] Quiroz JC, Laranjo L, Kocaballi AB, Berkovsky S, Rezazadegan D, Coiera E (2019). Challenges of developing a digital scribe to reduce clinical documentation burden. NPJ Digit Med.

[12] Greenhalgh T, Koh GCH, Car J (2020). Covid-19: a remote assessment in primary care. BMJ.

[13] Tobias M AI And Medical Diagnostics: Can A Smartphone App Detect Covid-19 From Speech Or A Cough? Forbes.

[14] Imran A, Posokhova I, Qureshi Haneya N, Masood Usama, Riaz Muhammad Sajid, Ali Kamran, John Charles N, Hussain Md Iftikhar, Nabeel Muhammad (2020). AI4COVID-19: AI enabled preliminary diagnosis for COVID-19 from cough samples via an app. Inform Med Unlocked.

[15] Dojchinovski D, Ilievski A, Gusev M (2019). Interactive home healthcare system with integrated voice assistant.

[16] Furst J Mayo Clinic introduces skill for Amazon’s Alexa about COVID-19. Mayo Clinic.

